# Characterization of *Shigella sonnei* Isolate Carrying Shiga Toxin 2–Producing Gene

**DOI:** 10.3201/eid2105.140621

**Published:** 2015-05

**Authors:** Outi Nyholm, Taru Lienemann, Jani Halkilahti, Sointu Mero, Ruska Rimhanen-Finne, Ville Lehtinen, Saara Salmenlinna, Anja Siitonen

**Affiliations:** National Institute for Health and Welfare, Helsinki, Finland (O. Nyholm, T. Lienemann, J. Halkilahti, R. Rimhanen-Finne, S. Salmenlinna, A. Siitonen);; Helsinki University Central Hospital Laboratory, Helsinki (S. Mero);; Central Hospital of Päijät-Häme, Lahti, Finland (V. Lehtinen)

**Keywords:** Shigella sonnei, Shiga toxin 2, STEC, diarrhea, virulence factors, bacteria, bacillary dysentery, shigellosis

**To the Editor:** *Shigella sonnei* causes a bacillary dysentery called shigellosis. Shiga toxins 1 (Stx1) and 2 (Stx2) are mainly produced by Shiga toxin–producing *Escherichia coli* (STEC), but Stx1 can also be produced by *S. dysenteriae* serotype 1 (*1*). Compared with STEC-producing Stx1, STEC-producing Stx2 has been reported to be more highly pathogenic and to be associated with hemolytic uremic syndrome (HUS) and hemorrhagic colitis (*2*); the association with HUS especially has been reported for subtypes Stx2a and Stx2c (*3*).

Stx-converting bacteriophages play a key role in expression of the *stx* gene in *E. coli* and in the lateral gene transfer between the bacteria (*4*). Although these bacteriophages have not been isolated from *S. dysenteriae* serotype 1, evidence suggests that the bacteria’s *stx* gene may be associated with a bacteriophage (*5*). The *stx_1_* gene, which is located in a bacteriophage, has previously been detected in *S. sonnei* (*6*). We describe an *S. sonnei* isolate with the *stx_2a_* gene.

In November 2013, 3 days after her return to Finland from a 2-week visit to relatives in southern Morocco, a 49-year-old woman was admitted to a hospital for bloody diarrhea, fever, and abdominal pain. Her symptoms began with watery diarrhea and fever 5 days before she returned home. The diarrhea ceased after a few days, but symptoms worsened, and diarrhea was visibly bloody after she returned to Finland. On admission, the patient had C-reactive protein and hemoglobin levels of 41 mg/L (reference value <3 mg/L) and 118 g/L (reference value 117–155 g/L), respectively; no thrombocytopenia was observed. No antimicrobial drugs were prescribed because STEC infection was clinically suspected. The patient responded well to intravenous fluid treatment and was discharged from the hospital 3 days after admission. At a follow-up visit 1 week later, she was asymptomatic and had a C-reactive protein level of 19 mg/L.

*Shigella* spp. was isolated from a feces sample obtained from the patient on illness day 8, and an STEC signal (based on the PCR-positive *stx_2_* gene) was detected from the same sample. A presumptive STEC strain was subsequently isolated. Both isolates were sent for confirmation and further typing to the Bacteriology Unit, National Institute for Health and Welfare, in Helsinki. The first isolate (FE109024) was confirmed as *S. sonnei* by matching of its biochemical profile to that of the reference strain ATCC 25931 (http://www.atcc.org/Products/All/25931.aspx; Technical Appendix Table) and agglutination by antiserum to *S. sonnei* (Denka Seiken, Tokyo, Japan). The agglutination by *S. sonnei* phase I antiserum indicated the smooth form of lipopolysaccharide. Atypical for *S. sonnei*, the isolate did not ferment mannitol (*7*). The isolate carried the genes encoding invasion plasmid antigen H, the invasion-associated locus, and invasion gene transcription regulator invE. The second isolate (FE109046) was initially sent to the National Institute for Health and Welfare as an STEC. However, FE109046 showed biochemical reactions in tubes and when using API 20E (bioMérieux, Durham, NC, USA) that were identical to reactions for isolate FE109024 after an overnight and 3-day incubation (Technical Appendix Table). Both isolates were nonmotile. FE109046 also agglutinated by *S. sonnei* polyvalent antiserum but was negative for *S. sonnei* phase I antiserum and positive for phase II antiserum, indicating the rough form of lipopolysaccharide. In contrast to FE109024, isolate FE109046 lacked the *Shigella*/enteroinvasive *E. coli*–specific *invE* gene but harbored the STEC-specific *stx_2_* gene. Neither isolate carried other STEC-associated genes. Subtyping of the *stx_2_* gene of isolate FE109046 was performed according to the published protocol (*8*); the isolate was confirmed to be subtype *stx_2a_*.

Genomic comparison of the 2 isolates was performed by using pulsed-field gel electrophoresis according to the standard protocol. The isolates showed 96% similarity. A 3-fragment difference suggests that 1 isolate was a variant of the other (Figure); the variant may have arisen through lysogenization with the phage.

**Figure:**
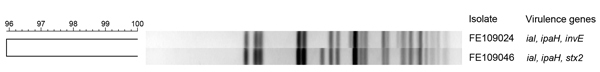
UPGMA dendrogram of *Xba*I restriction patterns of 2 *Shigella sonnei* isolates from a patient from Finland who became ill during a visit to Morocco. Genomic comparison of the 2 isolates was performed by using pulsed-field gel electrophoresis according to the standard protocol. The isolates showed 96% similarity, and a 3-fragment difference suggests that 1 isolate was a variant of the other. Scale bar represents % similarity. *ial*, invasion-associated locus; *ipaH*, invasion plasmid antigen H; *invE*, invasion gene transcription regulator invE; *stx_2_*, Shiga toxin 2.

The Shiga toxin subtype *stx_2a_* identified in isolate FE109046 is linked to severe human disease, including HUS (*3*). The role of Stx in shigellosis is unclear. Stx is not essential for cell invasion or lysis (*9*). In the previously reported case of *stx_1_*–positive *S. sonnei* infection, the patient’s symptoms were not severe, and diarrhea lasted 7 days (*6*).

*Shigella* spp. can harbor virulence genes found in *E. coli* because both are genetically defined as members of the same species (*10*). The *stx_2_*–positive *S. sonnei* may have emerged in the patient initially infected with a mannitol-negative *S. sonnei* that was subsequently lysogenized by transduction from a STEC co-infection or by a free *stx_2_* phage. *S. sonnei* isolates are not routinely examined for the production of Stx or *stx* genes in clinical laboratories, so the properties associated with STEC in *S. sonnei* isolates from patients remain undetected. *S. sonnei* with *stx_2a_* may have potential to cause severe disease, especially in children. This novel and remarkable virulence characteristic in *Shigella* spp. would affect diagnostics, infection control, and prevention.

Technical Appendix Characteristics of 2 isolates from a patient compared with those of a *Shigella sonnei* reference strain.
