# Supplementary material for: Characterization of Shigella sonnei Isolate Carrying Shiga Toxin 2–Producing Gene
Source: Emerg Infect Dis. 2015 May;21(5):891–2. doi: 10.3201/eid2105.140621 (PMC4412214; doi:10.3201/eid2105.140621)
Supplement: Technical Appendix — Characteristics of 2 isolates from a patient compared with those of a Shigella sonnei reference strain. [file 14-0621-Techapp-s1.pdf]

Article DOI: http://dx.doi.org/10.3201/eid2105.140621

Characterization of *Shigella sonnei* Isolate Carrying Shiga Toxin 2–Producing Gene

Technical Appendix

Technical AppendixTable. Characteristics of 2 isolates from a patient compared with those of a *Shigella sonnei* reference strain*

	Patient isolate		
Identification and typing tests	FE109024	FE109046	*S. sonnei* ATCC 25931
Sorbitol fermentation	–	–	–
Glucose fermentation	±†	±	±
Lactose fermentation	–	–	–
β-galactosidase production	+	+	+
Saccharose fermentation	–	–	–
Mannitol fermentation	–	–	±
Citrate fermentation	–	–	–
Inositol fermentation	–	–	–
Malonate fermention	–	–	–
Arabinose fermentation	+	+	+
Xylose fermentation	–	–	–
Dulcitol fermentation	–	–	–
Salicine fermentation	–	–	–
Growth on media containing potassium cyanide	–	–	–
Indole production	–	–	–
Urease production	–	–	–
Hydrogen sulfide production	–	–	–
Motility	–	–	–
API 20E test result	*Shigella sonnei* 89,2%‡	*Shigella sonnei* 89,2%§	*Shigella sonnei* 98,3%¶
API 20E code	1104002	1104002	1104102
S. sonnei polyvalent antiserum	+	+	+
S. sonnei phase I antiserum	+	–	±
S. sonnei phase II antiserum	–	+	±
stx_2	–	+	–
stx_2 subtype	Not applicable	stx_{2a}	Not applicable
ipaH	+	+	Not done
ial	+	+	Not done
invE	+	–	Not done
eaeA	–	–	Not done
hlyA	–	–	Not done
saa	–	–	Not done

**stx2*, Shiga toxin 2; *ipaH*, invasion plasmid antigen H; *ial*, invasion-associated locus; *invE*, invasion gene transcription regulator invE; *eaeA*, intimin; *hlyA*, enterohemolysin; *saa*, STEC autoagglutinating adhesin. *S. sonnei* ATCC 25931 forms 2 types of colonies: the smooth colony agglutinated by phase I antiserum, and the rough colony agglutinated by phase II antiserum.
†In glucose and mannitol test tubes, production of gas was also observed: ± = ferments carbohydrate, does not produce gas.‡ Test against mannitol 99%, rhamnose 75%.
§Test against mannitol 99%, rhamnose 75%.
¶Test against rhamnose 75%.
